# Effect of temperature on the biological cycle of *Aedes aegypti* under laboratory conditions

**DOI:** 10.17843/rpmesp.2024.413.13838

**Published:** 2024-08-30

**Authors:** Archi Alejandro Ruiz-Polo, Lourdes Viviana Barrera-Rivera

**Affiliations:** 1 Entomology Research and Training Center - CICE, Sub Regional Health Directorate Luciano Castillo Colonna, Sullana, Piura, Peru. Entomology Research and Training Center - CICE Sub Regional Health Directorate Luciano Castillo Colonna Sullana Piura Perú

To the editor. *Aedes aegypti* is the main vector of dengue virus (DENV), which is transmitted to humans by the bite of a previously infected female, i.e., a horizontal transmission route ^1^. However, molecular studies in the last decade have found that eggs collected during virus outbreaks were found to be infected, presuming transovarial and/or vertical transmission ^2^^,^^3^. Likewise, genetic variations are manifested in these epidemic scenarios, which then lead to the formation of subpopulations and subspecies with biological changes such as different levels of anthropophilia, resistance to insecticides and vectorial competition ^4^^,^^5^; in addition to interspecific relationships such as coexistence with other species of mosquitoes transmitting different infectious agents ^6^. Therefore, it is important to understand that the biological cycle of *Aedes aegypti* is completed in approximately 7 to 10 days, depending on the environmental temperature ^7^, which is altered by El Niño Southern Oscillation (ENSO) by influencing the geographical distribution of the mosquito ^8^^,^^9^. Besides, it is relevant to study their physiology, since future projections indicate that ENSO will become an extreme and frequent phenomenon due to climate change ^10^, so there is a need to experimentally investigate the biological cycle with eggs collected during dengue outbreaks, opting for analyses in microenvironments that emulate climatic scenarios at different temperatures.

We conducted an *in vitro*, quantitative and experimental research in which we evaluated the biological cycle of *Aedes aegypti* from 200 eggs collected during a dengue outbreak in 2023 in the district of Bellavista (4°53′27″S / 80°40′51″W) in the province of Sullana. Initially, we analyzed the eggs in a ZEISS Stemi DV4 model stereoscope in order to discard those dehydrated and with surface rupture, then we selected 100 eggs for treatment A and 100 eggs for treatment B. The eggs in the treatment A group were placed in a white plastic tray to which 500 milliliters of rested drinking water with 0.2 mg/L of residual chlorine were added; then the tray was exposed to an ambient temperature of 38.5 ± 1 °C and a humidity of 49.5 ± 2% for two days using an ILUMI EW-01 model heater in a closed isolated area.

Subsequently, we selected 20 L1 stage larvae from the first group of hatched eggs and exposed them to the same conditions for 10 consecutive days. At larval stages 1, 2, 3 and 4, larvae were fed daily with a mixture of chicken meal (purine) and commercial brewer’s yeast previously sieved on a No. 60 250 uM sieve. During these stages, water was replaced before each feeding, however, when changing to the pupal stage, the larvae were placed in white plastic cups with a tulle on the contour and 200 ml of rested water with 0.2 mg/L of residual chlorine. At this stage, they were no longer fed and water was not changed.

Treatment B group was under the same conditions as treatment A, however, the eggs were exposed to a different environmental temperature and humidity, 28.4 ± 4 °C and 64.7 ± 13%, respectively. Temperature and humidity were measured with a Taylor Light 1523 model thermohygrometer (water temperature was not measured), and residual chlorine with a DR900 model colorimeter (0.01 mg/L error). Egg hatching was evaluated every 12 hours. Data were routinely recorded in physical laboratory formats, and later analyzed in Excel version 2021 spreadsheets.

Regarding the results, 78% of the eggs under treatment A hatched in 1 day. With this treatment, the biological cycle took a minimum of 7 days and a maximum of 11 days, showing that 40% of adults were males, 20% were females and another 40% did not reach the adult stage (pupa) (Table 1). We also found that the larval stages lasted 1 day and the change from pupa to adult took less than 2 days (approximately 1.5 days). In contrast, 53% of treatment B eggs hatched in 2 days. With this treatment, the biological cycle took a minimum of 11 days and a maximum of 12 days, 15% of adults were females and 85% did not reach the adult stage (pupa) (Table 1). Similarly, the larval stages lasted more than 1 day and the change from pupa to adult took more than 2 days.

**Table 1:** Life cycle of *Aedes aegypti* by treatment.

Biological cycle	Treatment A ^a^	Treatment B ^b^
Egg hatching time	1 day	2 days
Minimum biological cycle	7 days	11 days
Maximum biological cycle	11 days	12 days
Analyzed eggs	100 (100%)	100 (100%)
Hatched eggs	78 (78%)	53 (53%)
Unhatched eggs	22 (22%)	47 (47%)
Analyzed larvae	20 (100%)	20 (100%)
Emerging male adults	8 (40%)	0 (0%)
Emerging female adults	4 (20%)	3 (15%)
Pupas	8 (40%)	17 (85%)

a Treatment with drinking water rested for 24 hours with 0.2 mg/L residual chlorine, mean ambient temperature of 38.5 ± 1 °C and relative humidity of 49.5 ± 2%, for 11 days.

b Treatment with drinking water rested for 24 hours with 0.2 mg/L of residual chlorine, average ambient temperature of 28.4 ± 4 °C and relative humidity of 64.7 ± 13%, for 12 days.

Our study is limited by the lack of multi-parameter equipment to measure complementary abiotic factors. Nevertheless, the obtained data are relevant in demonstrating the effect of temperature on the biological cycle of *Aedes aegypti*.

In conclusion, at a temperature of 38.5 ± 1 °C, humidity of 49.5 ± 2% and 0.2 mg/L of residual chlorine in water, the life cycle of *Aedes aegypti* is short and lasts a minimum of 7 days, mostly in males. This differs at 28.4 ± 4 °C, 64.7 ± 13% and 0.2 mg/L residual chlorine, where the life cycle takes a minimum of 11 days, mostly in females. This information is important for vector control strategies during climatic phenomena such as ENSO, since temperature, humidity and chlorine could be used as indicators of female abundance and potential dengue outbreaks.
