# Supplementary material for: Effect of temperature on the biological cycle of Aedes aegypti under laboratory conditions
Source: Rev Peru Med Exp Salud Publica. 2024 Aug 30;41(3):327–8. doi: 10.17843/rpmesp.2024.413.13838 (PMC11495923; doi:10.17843/rpmesp.2024.413.13838)
Supplement: Translation

# Translation: Efecto de la temperatura en el ciclo biológico del *Aedes aegypti* en condiciones de laboratorio

**Authors:** Archi Alejandro Ruiz-Polo, Lourdes Viviana Barrera-Rivera

**Affiliations:** 1 Centro de Investigación y Capacitación en Entomología - CICE, Dirección Sub Regional de Salud Luciano Castillo Colonna, Sullana, Piura, Perú.

Sr. Editor. *Aedes aegypti* es el principal vector del virus dengue (DENV), que es transmitido al ser humano por la picadura de una hembra previamente infectada, es decir, una vía de transmisión horizontal ^1^. Sin embargo, estudios moleculares de la última década, han hallado que huevos recolectados durante brotes del virus se encontraron infectados, presumiendo una transmisión transovárica y/o vertical ^2^^,^^3^. Así mismo, en estos escenarios epidémicos, se manifiestan variaciones genéticas que dan lugar a la formación de subpoblaciones y subespecies con cambios biológicos como los niveles de antropofilia, la resistencia a insecticidas y la competencia vectorial ^4^^,^^5^; además de relaciones interespecíficas como la coexistencia con otras especies de mosquitos transmisores de distintos agentes infecciosos ^6^. Por tanto, conociendo que el ciclo biológico de *Aedes aegypti* se completa aproximadamente entre 7 a 10 días, en función de la temperatura ambiental ^7^, que en escenarios que El Niño Oscilación del Sur (ENSO) se altera e influye en la distribución geográfica del mosquito ^8^^,^^9^; es relevante estudiar su fisiología, ya que proyecciones futuras señalan que ENSO se convertirá en un fenómeno extremo y de alta frecuencia debido al cambio climático ^10^, por lo que existe la necesidad de investigar experimentalmente el ciclo biológico con huevos recolectados durante brotes de dengue, optando por análisis en microambientes que emulen escenarios climáticos con temperaturas variables.

Se realizó una investigación *in vitro*, de enfoque cuantitativo y de diseño experimental en la que evaluamos el ciclo biológico del *Aedes aegypti* a partir de 200 huevos recolectados durante un brote de dengue del año 2023 en el distrito de Bellavista (4°53′27″S / 80°40′51″O) de la provincia de Sullana. Inicialmente, analizamos los huevos en un estereoscopio modelo ZEISS Stemi DV4 con el fin de descartar aquellos deshidratados y con ruptura superficial, luego seleccionamos 100 huevos para el tratamiento A y 100 huevos para el tratamiento B. En el tratamiento A, los huevos se colocaron en una bandeja blanca de plástico donde se añadió 500 mililitros de agua potable reposada con 0,2 mg/L de cloro residual, luego en un área aislada cerrada, la bandeja se expuso a una temperatura ambiental de 38,5 ± 1 °C y una humedad de 49,5 ± 2% durante dos días mediante un calefactor modelo ILUMI EW-01.

Posteriormente, del primer grupo de huevos eclosionados, seleccionamos 20 larvas en estadio L1 y las expusimos a las mismas condiciones durante 10 días seguidos. En los cambios de los estadios larvarios 1, 2, 3 y 4, las larvas se alimentaron diariamente con una mezcla de comida de gallina (purina) y levadura de cerveza comercial previamente tamizada en una criba N.º 60 de 250 uM. Durante estos estadios, el recambio de agua se realizó antes de cada alimentación, sin embargo, al cambiar al estadio de pupa, estas se depositaron en vasos de plástico blanco con un tul en el contorno y 200 ml de agua reposada con 0,2 mg/L de cloro residual. En este estadio, ya no se alimentó y no se realizó recambio de agua.

En cuanto al tratamiento B, este se encontró en las mismas condiciones que el tratamiento A, sin embargo, los huevos se expusieron a una temperatura ambiental y una humedad distinta, 28,4 ± 4 °C y 64,7 ± 13%, respectivamente. La temperatura y humedad se midieron con un termohigrómetro modelo Taylor Light 1523 (la temperatura del agua no se midió), y el cloro residual con un colorímetro modelo DR900 (error de 0,01 mg/L). La eclosión de huevos la evaluamos cada 12 horas. El registro de datos se realizó en formatos físicos de laboratorio de forma rutinaria, posteriormente se analizaron en hojas de cálculo en Excel versión 2021.

Con respecto a los resultados, en el tratamiento A la eclosión de huevos se observó en 1 día y en un 78%. En este tratamiento el ciclo biológico tardó 7 días como mínimo y 11 días como máximo, evidenciándose que el 40% de adultos fueron machos, el 20% fueron hembras y otro 40% no alcanzó el estadio de adulto (pupa) (Tabla 1). También, se observó que los estadios larvarios duraron 1 día y el cambio de pupa a adulto menos de 2 días (1,5 días aproximadamente). En contraste, en el tratamiento B la eclosión de huevos se observó en 2 días y en un 53%. En este tratamiento el ciclo biológico tardó 11 días como mínimo y 12 como máximo, hallándose que el 15% de adultos fueron hembras y que el 85% no alcanzó el estadio adulto (pupa) (Tabla 1). De igual manera, los estadios larvarios duraron más de 1 día y el cambio de pupa a adulto más de 2 días.

**Tabla 1:** Ciclo biológico de *Aedes aegypti* por tratamiento evaluado.

Ciclo biológico	Tratamiento A ^a^	Tratamiento B ^b^
Tiempo de eclosión de huevos	1 día	2 días
Ciclo biológico mínimo	7 días	11 días
Ciclo biológico máximo	11 días	12 días
Huevos analizados	100 (100%)	100 (100%)
Huevos eclosionados	78 (78%)	53 (53%)
Huevos no eclosionados	22 (22%)	47 (47%)
Larvas analizadas	20 (100%)	20 (100%)
Adultos machos emergentes	8 (40%)	0 (0%)
Adultos hembras emergentes	4 (20%)	3 (15%)
Pupas	8 (40%)	17 (85%)

a Tratamiento con agua potable reposada por 24 horas con 0,2 mg/L de cloro residual, temperatura ambiental media de 38,5 ± 1 °C y humedad relativa de 49,5 ± 2%, durante 11 días.

b Tratamiento con agua potable reposada por 24 horas con 0,2 mg/L de cloro residual, temperatura ambiental media de 28,4 ± 4 °C y humedad relativa de 64,7 ± 13%, durante 12 días.

El presente estudio posee la limitación de la falta de equipos multiparametros para medir factores abióticos complementarios. No obstante, los datos obtenidos son relevantes al evidenciar el efecto de la temperatura en el ciclo biológico del *Aedes aegypti*.

En conclusión, a una temperatura de 38,5 ± 1 °C, una humedad de 49,5 ± 2% y 0,2 mg/L de cloro residual en agua, el ciclo biológico de *Aedes aegypti* es corto en un mínimo de 7 días, siendo el macho el adulto de mayor número. Esto difiere a 28,4 ± 4 °C, 64,7 ± 13% y 0,2 mg/L de cloro residual, donde el ciclo biológico es largo en un mínimo de 11 días, siendo la hembra la de mayor número. Esta Información es importante para estrategias del control vectorial durante fenómenos climáticos como ENSO, ya que la temperatura, humedad y el cloro, podrían usarse como indicadores de la abundancia de hembras y de potenciales brotes de dengue.
